# Supplementary material for: Causal Relationship between Aging and Anorexia Nervosa: A White-Matter-Microstructure-Mediated Mendelian Randomization Analysis
Source: Biomedicines. 2024 Aug 16;12(8):1874. doi: 10.3390/biomedicines12081874 (PMC11351342; doi:10.3390/biomedicines12081874)
Supplement: Supplementary file 1 [file biomedicines-12-01874-s001.zip › Supplementary Figure/Figure S4/Singe SNP plot of MR analysis for TL to ubm-b-1651.pdf]

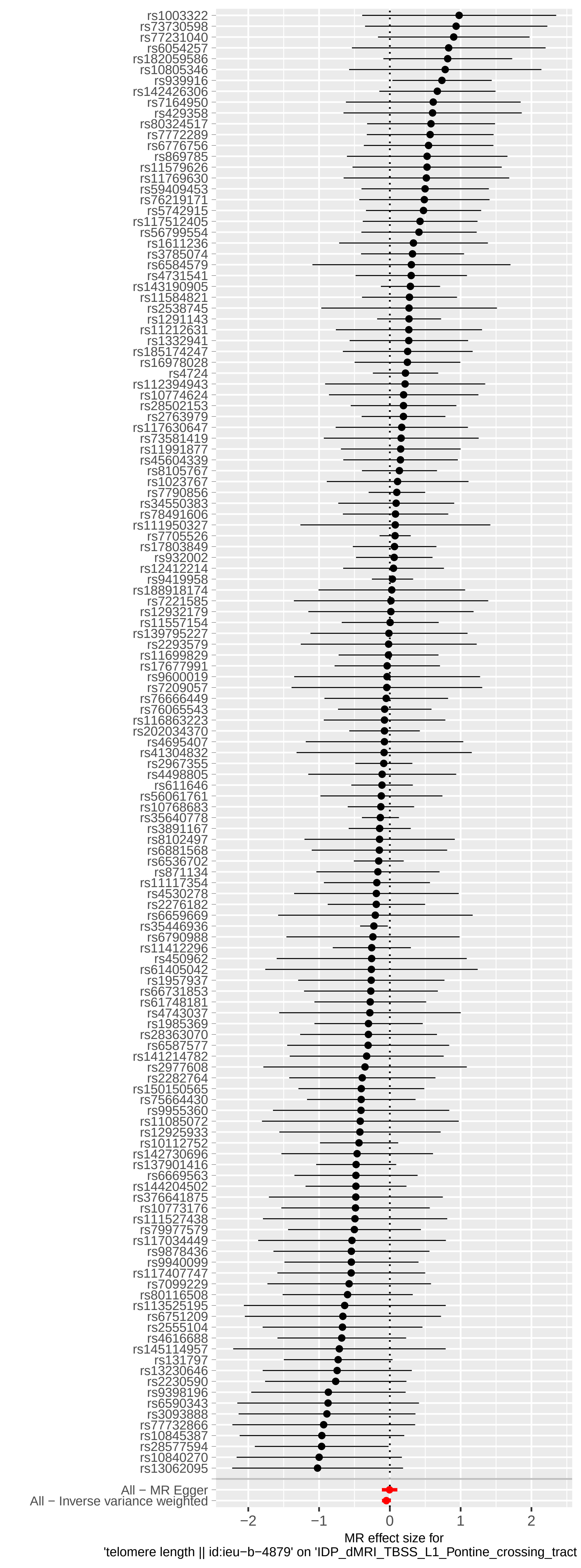
rs1003322
rs73730598
rs77231040
rs6054257
rs182059586
rs10805346
rs939916
rs142426306
rs7164950
rs429358
rs80324517
rs7772289
rs6776756
rs869785
rs11579626
rs11769630
rs59409453
rs76219171
rs5742915
rs117512405
rs56799554
rs1611236
rs3785074
rs6584579
rs4731541
rs143190905
rs11584821
rs2538745
rs1291143
rs11212631
rs1332941
rs185174247
rs16978028
rs4724
rs112394943
rs10774624
rs28502153
rs2763979
rs117630647
rs73581419
rs11991877
rs45604339
rs8105767
rs1023767
rs7790856
rs34550383
rs78491606
rs111950327
rs7705526
rs17803849
rs932002
rs12412214
rs9419958
rs188918174
rs7221585
rs12932179
rs11557154
rs139795227
rs2293579
rs11699829
rs17677991
rs9600019
rs7209057
rs76666449
rs76065543
rs116863223
rs202034370
rs4695407
rs41304832
rs2967355
rs4498805
rs611646
rs56061761
rs10768683
rs35640778
rs3891167
rs8102497
rs6881568
rs6536702
rs871134
rs11117354
rs4530278
rs2276182
rs6659669
rs35446936
rs6790988
rs11412296
rs450962
rs61405042
rs1957937
rs66731853
rs61748181
rs4743037
rs1985369
rs28363070
rs6587577
rs141214782
rs2977608
rs2282764
rs150150565
rs75664430
rs9955360
rs11085072
rs12925933
rs10112752
rs142730696
rs137901416
rs6669563
rs144204502
rs376641875
rs10773176
rs111527438
rs79977579
rs117034449
rs9878436
rs9940099
rs117407747
rs7099229
rs80116508
rs113525195
rs6751209
rs2555104
rs4616688
rs145114957
rs131797
rs13230646
rs2230590
rs9398196
rs6590343
rs3093888
rs77732866
rs10845387
rs28577594
rs10840270
rs13062095
All − MR Egger
All − Inverse variance weighted
−2
−1
0
1
2
MR effect size for
'telomere length || id:ieu−b−4879' on 'IDP_dMRI_TBSS_L1_Pontine_crossing_tract'
